# Awareness of Diabetic Retinopathy Among Patients With Type 2 Diabetes Mellitus in Primary Healthcare Centers in Madinah, Saudi Arabia: A Cross-Sectional Study

**DOI:** 10.7759/cureus.49718

**Published:** 2023-11-30

**Authors:** Muayad S Albadrani, Ahmed M Alrehaili, Sultan H Alahmadi, Abdulqader A Aljazaeri, Osama F Almaghthawi, Saif N Alanazi, Osamah A Alrehaili

**Affiliations:** 1 Department of Family and Community Medicine, Taibah University, Medina, SAU; 2 College of Medicine, Taibah University, Medina, SAU

**Keywords:** family medicine, ophthalmology, awareness, screening, diabetic retinopathy, madinah, saudi arabia

## Abstract

Background

The prevalence of diabetes mellitus (DM) in Saudi Arabia is among the highest in the Middle East and North Africa (MENA) regions. Various complications of DM can cause problems in the long term. One of the most prevalent microvascular problems and the primary cause of blindness is diabetic retinopathy (DR), and a significant proportion of the population with diabetes eventually develop diabetes retinopathy. Recognizing and understanding DR may be crucial for patients in identifying and averting this complication.

Objectives

The objective of this atudy is to assess the awareness of DR among patients with type 2 DM at primary healthcare centers in Madinah, Saudi Arabia.

Methods

This cross-sectional study involved a survey of patients with diabetes who attended Madinah primary care clinics between August and September 2023. The study was conducted in Madinah, Saudi Arabia, from May to November 2023.

Results

A total of 240 patients participated with a median age of 49.7 years and a gender distribution of 121 (50.4%) men. Overall, less than half of patients had a fair level of knowledge (47.1%) and a good level of knowledge (42.1%) about DR, whereas 10.8% had poor knowledge. Physicians were the primary source of information for patients, followed by the internet, family, and friends. Higher levels of education, diabetes that had been present for a longer period, and regular eye exams were associated with better understanding. This study emphasizes the importance of improving patient knowledge and awareness of DR.

Conclusions

We observed a high level of awareness of DR among participants. Furthermore, higher awareness was associated with longer disease duration and compliance with diabetes treatment.

## Introduction

Diabetes mellitus (DM) is a dangerous chronic disorder in which the body does not create enough insulin or does not use the insulin that it produces adequately. Although type 1 diabetes most frequently affects children, it can affect individuals at any age. This form of diabetes cannot be prevented, and insulin is necessary for patients with type 1 diabetes to survive. By contrast, most diabetes cases worldwide, more than 90%, are type 2, making it the most prevalent form of the disease [1]. According to the International Diabetes Federation, the prevalence of DM in Saudi Arabia is 18.7%, one of the highest rates in the Middle East and North Africa (MENA) region [1]. Another study predicted that this percentage will double by 2030 [2].

Numerous acute and long-term complications can result from uncontrolled diabetes. Furthermore, retinopathy, neuropathy, and nephropathy are chronic microvascular complications, whereas cerebrovascular disease, coronary artery disease, and peripheral arterial disease are chronic macrovascular complications. Diabetes is a major contributor to cardiovascular disease, blindness, kidney failure, and lower limb amputations [3]. Health education is a potent tool for managing chronic health issues such as diabetes, as demonstrated in other nations [4]. A better understanding and awareness of DM among the public would help improve community health outcomes. Increasing patients’ knowledge of DM could maximize their quality of life and enhance their medication habits, thereby enabling them to achieve optimal health benefits and delay the onset of long-term complications. Additionally, education is crucial to support the dietary and psychological needs of families of patients with DM and assist them in making necessary lifestyle changes [4].

Diabetic retinopathy (DR) is the most prevalent microvascular complication of DM; it is also the most common cause of blindness. Worldwide, the prevalence of DR has significantly increased [5]. DR affects the retinal blood flow and impairs the transport of nutrients and oxygen to the retina; thus, the retina’s high metabolic needs are not met [6]. The prevalence of DR in Saudi Arabia’s central region is 14.8% [7], although studies in other regions of Saudi Arabia have revealed a prevalence between 27.8% and 36.4% [8,9]. Age, male gender, high blood pressure, diabetes duration, diabetic foot ulcer, diabetic foot amputation, fasting blood sugar, serum total cholesterol, serum triglycerides, and HbA1c were associated with DR [10].

Research has demonstrated that young patients, mostly less educated females, have a higher incidence of DR because they lack knowledge of this complication [11]. DR may go unnoticed until patients have a considerable loss of vision, and some patients may skip their annual eye exams. Skipping the eye exam won’t result in DR but could result in it presenting at a later (and more difficult to manage) stage [12,13]. Early screening and intervention can prevent blindness; conversely, poor adherence to routine DR screening can negatively impact patients’ quality of life and place strain on healthcare systems. This poor adherence can result from a lack of awareness among patients and primary care physicians [14]. Other factors, such as limited access to ophthalmologists, time, money, and transportation, may also have an effect on patient adherence in rural areas [15].

Research evaluating awareness of DR among patients with T2DM in Madinah, Saudi Arabia, is currently scarce. In light of the worrying rise in T2DM cases in Saudi Arabia, we conducted this study in primary health centers in Madinah to determine the level of awareness of DR among patients with T2DM.

## Materials and methods

This cross-sectional observational analytical study was conducted in Madinah, Saudi Arabia, using a predesigned questionnaire from a study conducted in Riyadh, Saudi Arabia (20). The study utilized a convenient non-probability sampling method of patients with early T2DM aged 18-85 years who attended for follow-up or picked up their medications in primary health care centers in Madinah from August to September 2023. Patients with type 1 DM and patients younger than 18 years old with diabetes were excluded from the study. The four sections of the predesigned questionnaire were demographic characteristics, awareness questions, compliance questions, and barrier questions. The questionnaire was composed of 22 questions: five of them were about DR knowledge, three questions were about screening, and two were about prevention and treatment. Before recruitment, participants were given details about the study’s objectives, including its duration and confidentiality. They were also informed that their data would be used for study-related purposes, but their identities would be kept confidential, to which they agreed. The Taibah University Scientific Research Ethics Committee approved the project (study ID: COPTU-REC-74-20230810). 

Statistical analysis

To analyze patients’ responses to knowledge questions and statements, a score of 1 was assigned for correct answers and 0 for incorrect or “don’t know” responses. The overall score and percentage for each participant were then computed. Patients who scored 50% or lower were considered to have poor knowledge, those who scored 50-75% were considered to have fair knowledge, and those who scored more than 75% were considered to have strong knowledge.

The IBM SPSS Statistics, version 28.0 (IBM Corp., Armonk, NY) was used for data entry and statistical analysis. Frequency and percentage were used to describe the data for categorical variables, whereas the mean and standard deviation (SD) were used for numerical continuous variables. The chi-square test was used to determine differences and/or associations between categorical variables, and two-sample independent t-tests were used to compare the arithmetic mean of continuous variables between two groups. A p-value of 0.05 was used to determine statistical significance for all analyses.

## Results

Demographic characteristics

The study included 240 patients. Table 1 lists their demographic features. Their ages ranged from 22 to 85 years (median, 49.7 years; SD, 12.4 years). In all, 121 participants were male (50.4%), and 155 patients (64.6%) had a college education.

**Table 1:** Demographics of individuals with type 2 diabetes (n = 240)

	Frequency	Percentage
Sex		
Male	121	50.4
Female	119	49.6
Age (years)		
Range	22–85	
Mean ± SD	49.7 ± 12.4	
Educational level		
No education	10	4.2
Elementary school	15	6.3
High school	60	25.0
University	155	64.6

Diabetes history and DR-related practices

In all, 216 (90%) patients reported a family history of diabetes, and 110 (45.8%) patients had a disease duration of over 10 years. The age at diabetes diagnosis ranged from 15 to 67 years (mean, 38.4; SD, 11.5 years). However, 170 (70.8%) patients reported a good level of blood sugar control, and 137 (57.1%) patients reported that their vision was affected by diabetes. Approximately one-third of patients, i.e., 81 (33.8%), reported that an ophthalmologist had referred them for a retinal and vision exam. Most patients reported that they adhered to diabetes medications and checked their blood sugar at home (84.2% and 84.6%, respectively). Of the included patients, 116 (48.3%) reported that they checked their blood sugar at home daily, and 87 (36.3%) patients stated that they had recently undergone a retinal examination by an ophthalmologist (Table 2).

**Table 2:** Diabetic retinopathy-related history and practices in patients with type 2 diabetes (n = 240)

Variables	Frequency	Percentage
Family history of diabetes		
No	24	10.0
Yes	216	90.0
Duration of diabetes (years)		
≤5	67	27.9
6–10	63	26.3
>10	110	45.8
Age at diagnosis of diabetes		
Range	15–67	
Mean ± SD	38.4 ± 11.5	
Blood sugar control level		
Good	170	70.8
Not good	70	29.2
Vision affection due to diabetes		
No	103	42.9
Yes	137	57.1
Referral from ophthalmologist to do a retinal and vision exam		
Referral from a therapist	81	33.8
Patient himself is self-conscious of the dangers of the diabetic retina	106	44.2
Not yet reviewed	53	22.0
History of taking diabetes treatment fully and correctly		
No	38	15.8
Yes	202	84.2
Checking blood sugar at home		
No	37	15.4
Yes	203	84.6
Frequency of checking blood sugar at home among type II diabetic patients		
No	37	15.4
Once monthly	11	4.6
Once weekly	35	14.6
Daily	116	48.3
When feeling unwell	41	17.1
History of seeing the ophthalmologist for a retinal examination		
During the past 6 months	87	36.3
During the past year	106	44.1
Not reviewed	47	19.6

**Figure 1:**
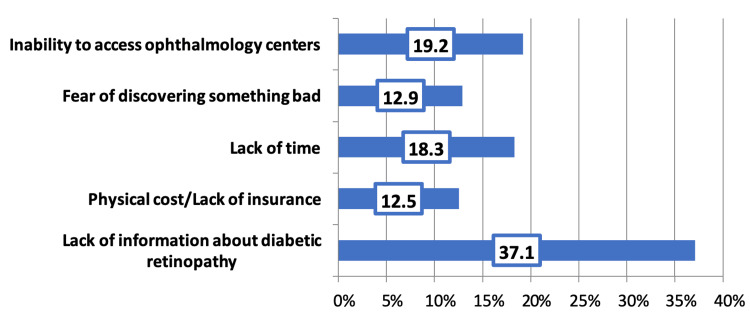
Reasons for not undergoing an early retinal vision exam

Lack of knowledge regarding DR (37.1%), difficulty accessing ophthalmology facilities (19.2%), and lack of time (18.3%) were the most cited barriers to receiving an early retinal vision exam (Figure 1).

Knowledge about DR

Most patients knew that diabetes could affect their retinas, i.e., 218 (90.8), that retinal exams are necessary for someone with diabetes, i.e., 221 (92.1%), and that controlling blood sugar levels can reduce the risk of DR, i.e., 211 (87.9%). Additionally, 173 (72.1%) patients knew that DR due to diabetes can lead to blindness. However, only 100 (41.7%) patients knew that laser treatment for DR does not improve vision but rather reduces its deterioration, and 60 (25%) patients knew that DR screening is required every year (Table 3). 

**Table 3:** Responses of patients with type II diabetes to knowledge questions regarding diabetic retinopathy

Questions	Correct answers		
	Response	No.	%
Did you know that diabetes affects the retina?	Yes	218	90.8
Did you know that diabetic retinopathy due to diabetes leads to blindness?	Yes	173	72.1
Did you know that controlling your blood sugar level can reduce your risk of diabetic retinopathy?	Yes	211	87.9
Do you think a retinal exam is necessary when you have diabetes?	Yes	221	92.1
How much time do you think would be required for your diabetes retinopathy screening?	Every year	60	25.0
Did you know that laser treatment for diabetic retinopathy does not improve vision but rather reduces its deterioration?	Yes	100	41.7

**Figure 2:**
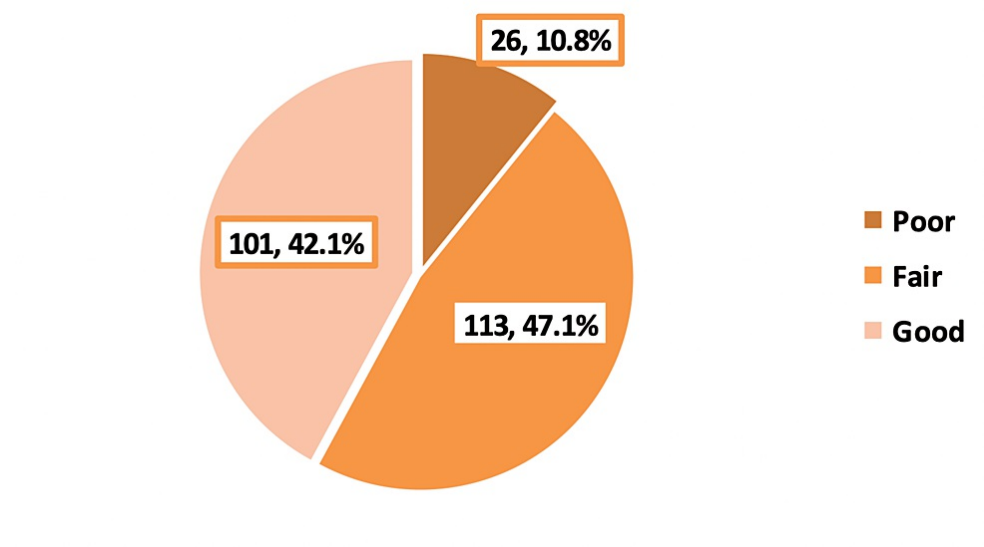
Overall level of knowledge about diabetic retinopathy among patients with type 2 diabetes

Overall, 101 (42.1%) patients had a good level of knowledge about DR, whereas 26 (10.8%) patients had poor knowledge, as illustrated in Figure 2. 

**Figure 3:**
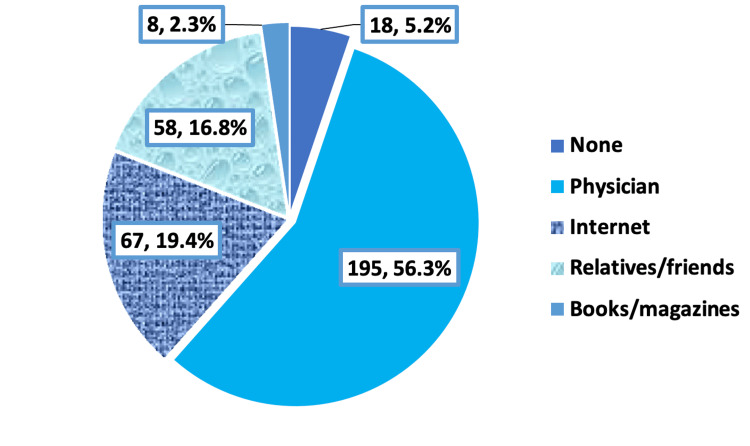
Main source of information about diabetic retinopathy among patients with type 2 diabetes

The main sources of information about DR were physicians, i.e., 195 (56.3%), followed by the internet, i.e., 67 (19.4%), and relatives/friends, i.e., 58 (16.8%); 18 (5.2%) of patients had no source of information about DR (Figure 3).

Patients with a longer duration of the disease (>10 years) were more likely to have a good level of knowledge about DR than those with a disease duration of five years or less (51.9% vs. 29.9%, p = 0.006). Patients diagnosed at a younger age were more likely to have good knowledge about DR than those diagnosed at an older age (mean ± SD age at diagnosis of those with good knowledge was 36.3 ± 11.4 years; mean ± SD age at diagnosis of those with poor knowledge was 39.0 ± 12.8 years). However, this difference did not quite reach statistical significance (p = 0.051). Patients who were self-educated about the dangers of DR were more likely than those who were referred by a therapist to have a good level of DR knowledge (50.9% vs. 30.9%, p = 0.036). Patients who reported taking diabetes medication correctly had a higher level of knowledge about DR compared with those who were not fully compliant with treatment (45.5% vs. 23.7%, p < 0.001). More than half (52.8%) of patients seen by ophthalmologists during the past year had a good level of knowledge about DR. In contrast, only 29.9% of those who were seen by ophthalmologists during the past six months had a good level of DR knowledge (p = 0.005). Patients who obtained their information from books/magazines, i.e., 120 (50.0%); physicians, i.e., 116 (48.3%); or relatives and friends, i.e., 116 (48.3%), were more likely than those who obtained their information from the internet (37.3%) to have a good level of knowledge about DR (p < 0.001; Table 4).

**Table 4:** Factors associated with knowledge about diabetic retinopathy among patients with type 2 diabetes *Chi-square test Two-sample independent t-test

Variables	Level of knowledge about diabetic retinopathy			p-value
	Poor N = 26 N (%)	Fair N = 113 N (%)	Good N = 101 N (%)	
Sex				
Male (n = 121)	14 (11.6)	59 (48.7)	48 (39.7)	0.739*
Female (n = 119)	12 (10.1)	54 (45.4)	53 (44.5)	
Age (years)				
Mean ± SD	45.4 ± 13.0	50.5 ± 12.1	50.0 ± 12.4	0.158•
Educational level				
Uneducated (n = 10)	0 (0.0)	7 (70.0)	3 (30.0)	0.369*
Elementary school (n = 15)	1 (6.7)	9 (60.0)	5 (33.3)	
High school (n = 60)	7 (11.7)	32 (53.3)	21 (35.0)	
University (n = 155)	18 (11.6)	65 (41.9)	72 (46.5)	
Family history of diabetes				
No (n = 24)	1 (4.2)	12 (50.0)	11 (45.8)	0.540*
Yes (n = 216)	25 (11.6)	101 (46.8)	90 (41.7)	
Duration of diabetes (years)				
≤5 (n = 67)	13 (19.4)	34 (50.7)	20 (29.9)	0.006*
6–10 (n = 63)	8 (12.7)	31 (49.2)	24 (38.1)	
>10 (n = 110)	5 (4.5)	48 (43.6)	57 (51.9)	
Age at diagnosis of diabetes				
Mean ± SD	39.0 ± 12.8	40.1 ± 11.1	36.3 ± 11.4	0.051•
Blood sugar control level				
Good (n = 170)	15 (8.8)	78 (45.9)	77 (45.3)	0.151*
Not good (n = 70)	11 (15.7)	35 (50.0)	24 (34.3)	
Vision affected by diabetes				
No (n = 103)	11 (10.7)	46 (44.7)	46 (44.7)	0.773*
Yes (n = 137)	15 (10.9)	67 (48.9)	55 (40.1)	
Referral from ophthalmologist for a retinal and vision exam				
Referral from a therapist (n = 81)	14 (17.3)	42 (51.8)	25 (30.9)	0.036*
Patient self-educated about the dangers of diabetic retinopathy (n = 10)	7 (6.6)	45 (42.5)	54 (50.9)	
Not yet reviewed (n =53)	5 (9.4)	26 (49.1)	22 (41.5)	
History of taking diabetes treatment fully and correctly				
No (n = 38)	12 (31.6)	17 (44.7)	9 (23.7)	<0.001*
Yes (n = 202)	14 (6.9)	96 (47.6)	92 (45.5)	
Checking blood sugar at home				
No (n = 37)	6 (16.2)	18 (48.6)	13 (35.1)	0.429*
Yes (n = 203)	20 (9.9)	95 (46.8)	88 (43.3)	
Frequency of checking blood sugar at home among patients with type 2 diabetes				
No (n = 37)	6 (16.2)	18 (48.6)	13 (35.1)	0.137*
Once monthly (n = 11)	1 (9.1)	5 (45.5)	4 (45.4)	
Once weekly (n = 35)	5 (14.3)	12 (34.3)	18 (51.4)	
Daily (n = 116)	6 (5.2)	57 (49.1)	53 (45.7)	
When feeling unwell (n = 41)	8 (19.5)	21 (51.2)	12 (29.3)	
History of seeing an ophthalmologist for a retinal examination				
During the past 6 months (n = 87)	16 (18.4)	45 (51.7)	26 (29.9)	0.005*
During the past year (n = 106)	6 (5.7)	44 (41.5)	56 (52.8)	
Not reviewed (n = 47)	4 (8.5)	24 (51.1)	19 (40.4)	
Main source of information				
None (n = 18)	10 (55.6)	7 (38.9)	1 (5.6)	<0.001
Physician (n = 89)	5 (5.6)	41 (46.1)	43 (48.3)	
Internet (n = 67)	2 (3.0)	40 (59.7)	25 (37.3)	
Relatives/friends (n =58)	8 (13.8)	22 (37.9)	28 (48.3)	
Books/magazines (n =8)	1 (12.5)	3 (37.5)	4 (50.0)	

## Discussion

In the present study, the most common reason for not undergoing an early eye exam for DR was lack of information about DR; by contrast, a study in Taif showed that the main reason for not undergoing early screening of DR was patients’ inability to access ophthalmology clinics [16]. 

In our study, 216 (90%) of patients reported a family history of diabetes; this percentage was higher than that in two other studies conducted in Riyadh, Saudi Arabia, in which 70% and 79.8% of patients had a family history of diabetes [17,18]. In addition, the mean age at diagnosis was 38.4 ± 11.5 years, whereas it was 39.88 ± 15.6 in another study [18].

The age and duration of the disease are important factors in the development of DR. In this study, 110 (45.8%) of participants had a disease duration of less than 10 years. Patients who have a disease duration of >10 years were more likely to have a good level of knowledge about DR (51.9%) than those with a disease duration of five years or less (29.9%), similar to a study in Taif, which shows that 46.3% of participants with good knowledge who have T2D > 10 years were compared to the 28.1% of participants who have T2D < 5 years [16]. The development of DR and other complications is influenced by glycemic control [19]. In our study, 170 (70.8%) patients reported good levels of blood glucose control, and 116 (48.3%) patients checked their blood sugar levels at home daily. However, 137 (57.1%) patients reported that they had vision problems due to diabetes. Other studies have reported different percentages of 47.1% and 16% [16-19]. Approximately 44% of patients had obtained a retinal and vision exam because of their awareness of the dangers of DR, whereas 81 (33.8%) patients were referred for the exam by a therapist. Another study showed that 55.1% of patients were referred to an ophthalmologist by a GP, and 16.7% of patients sought an ophthalmology exam independently [18]. It is essential to keep in mind that a delay in the referral to an ophthalmologist can impact a patient’s quality of life [20]. Although our study found that 218 (90.8%) patients were aware of the effects of diabetes on the retina, only 87 (36.3%) patients had seen an ophthalmologist for a retinal examination in the previous six months, and 106 (44.1%) patients had seen an ophthalmologist in the past year. Similarly, another study found that only 69.5% of patients regularly visited an ophthalmologist, even though 98% of patients were aware that diabetes affects the retina [21].

Most participants in our study were aware that having diabetes might damage their retina, i.e., 218 (90.8%), that having diabetes necessitates a retinal exam, i.e., 221 (92.1%), and that lowering blood sugar levels can lessen the risk of DR, i.e., 211 (87.9%). In addition, 173 (72.1%) patients correctly identified the connection between DR and blindness. This supports the results of AlHargan et al., who found that 247 (88%) participants knew that DM could impair the retina, 214 (76%) patients knew that blood sugar control lowers the risk of DR, and 186 (66%) patients knew that DR could result in blindness [17].

This study found that a high percentage of patients were aware of DM’s impact on the eyes, similar to previous research conducted in Saudi Arabia in Hail and in Al Jouf and Jeddah, in which 76% and 83% of patients were aware of DM’s effects on the eyes, respectively [22,23]. Regional studies in Oman [24], Jordan [25], and Turkey [26] also showed that a high percentage of patients were aware that DM could affect the eyes (93%, 88%, and 88%, respectively), similar to the percentage found in the current study (88%). A large percentage of patients in Switzerland (96%) [27] and Malaysia (86%) [6] also showed high awareness; however, a study in rural Tamil Nadu, India, revealed a low degree of awareness (74%) [28] about DR and DM.

Only 100 (41.7%) of participants in the current study were aware that laser treatment for DR does not enhance eyesight but only slows down vision deterioration; similarly, 195 (56.3%) of participants in a primary healthcare research study conducted in a security force hospital in Riyadh, Saudi Arabia, believed that laser treatment does not enhance vision [18]. 

In the present study, patients with a disease duration of over 10 years were more likely to be more knowledgeable about DR than those with a disease duration of five years or less. Similar findings were reported by studies conducted in Turkey [17] and Taif [16]. Patients who were self-educated about the dangers of DR were more likely than those who were referred by a therapist to have a good level of DR knowledge. Patients who reported taking diabetes medication fully and correctly had a higher level of knowledge about DR than those who were not fully compliant with their treatment. A study conducted at a security force hospital in Riyadh, Saudi Arabia, reported similar findings [18].

Strengths and limitations

The present study has several strengths. First, it involved a detailed investigation of awareness about DR among individuals with diabetes and assessed their overall knowledge of DM. In addition, because we included patients from primary care facilities, our results likely reflect the actual level of awareness and practices among patients with diabetes. However, this study also has several limitations. Because the study was conducted in only one Saudi Arabian city, the generalizability of the results may be limited. Additionally, the non-probability sampling method may be subjected to selection bias. Furthermore, the data regarding DR knowledge and disease duration were gathered via self-reported responses to questionnaire items rather than from medical records, and thus, some patients may have over-reported. 

Recommendations

Future research should overcome the limitations mentioned above and consider exploring additional factors that could affect patients’ understanding and awareness of DR. Policymakers should create educational programs to raise the awareness of DR among individuals with diabetes and stress the importance of routine eye exams. Primary care physicians should help patients understand how DM and DR affect their health. To lessen the long-term effects of DR, family medicine physicians should provide timely advice and referrals for patients with diabetes. Comprehensive treatment programs and continuous illness progression monitoring will be possible, thanks to the experts and family physicians’ cooperative approach.

## Conclusions

Most participants in the current study had a high level of knowledge regarding DR. Participants with longer disease duration and adherence to diabetes treatment also had higher awareness levels. Furthermore, patients who were aware of the hazards associated with DR, those who were diagnosed at a younger age, and those who had seen an ophthalmologist in the past year were more likely to have a strong understanding of DR. Policymakers should develop educational programs to improve patients’ knowledge of DR, encourage routine eye exams, and offer timely counseling. A collaborative approach between professionals and family physicians is necessary to create comprehensive treatment programs.
